# Integration of Horse Manure Vermicompost Doses and Arbuscular Mycorrhizal Fungi to Improve Fruit Quality, and Soil Fertility in Tomato Field Facing Drought Stress

**DOI:** 10.3390/plants13111449

**Published:** 2024-05-23

**Authors:** Soufiane Lahbouki, Abeer Hashem, Ajay Kumar, Elsayed Fathi Abd_Allah, Abdelilah Meddich

**Affiliations:** 1 “Physiology of Abiotic Stresses” Team, Research Unit Labeled CNRST (Centre AgroBiotech-URL-CNRST-05), Center of Agrobiotechnology and Bioengineering, Cadi Ayyad University, Marrakech 40000, Morocco; 2 Laboratory of Agro-Food, Biotechnologies and Valorization of Plant Bioresources (AGROBIOVAL), Department of Biology, Faculty of Sciences Semlalia, Cadi Ayyad University, Marrakesh 40000, Morocco; 3 Botany and Microbiology Department, College of Science, King Saud University, P.O. Box 2460, Riyadh 11451, Saudi Arabia; habeer@ksu.edu.sa; 4 Amity Institute of Biotechnology, Amity University, Sector-125, Noida 201313, Uttar Pradesh, India; ajaykumar_bhu@yahoo.com; 5 Plant Production Department, College of Food and Agricultural Sciences, King Saud University, P.O. Box 2460, Riyadh 11451, Saudi Arabia; eabdallah@ksu.edu.sa

**Keywords:** tomato, drought, organic amendments, arbuscular mycorrhizal fungi, fruit quality, soil health, sustainable agriculture

## Abstract

Climate change poses major challenges for agriculture in arid and semi-arid regions, with drought conditions severely affecting water-intensive crops such as tomatoes. This study evaluates the efficacy of organic amendments, derived from horse manure, and arbuscular mycorrhizal fungi (AMF) on enhancing tomato (*Solanum lycopersicum* L.) fruit quality and soil health under semi-arid field conditions. The experimental design included two irrigation regimes (well-watered and drought stress) and two levels of vermicompost application (C1 5 t ha^−1^ and C2 10 t ha^−1^), applied individually or in combination with AMF. The results indicate that drought stress reduced tomato fruit growth and yield, while osmoprotectant accumulation, antioxidant enzyme activity, and bioactive compound levels increased, and the 2,2-diphenyl-1-picrylhydrazyl radical scavenging activity of tomato fruit also increased. Notably, the biostimulants application, especially (C1+AMF), counteracted the adverse effects of drought, compared to the control, by significantly enhancing fruit yields (60%), as well as increasing ascorbic acid levels (59%) and free amino acids content (90%). These treatments also improved the activity of bioactive compounds and nutrient uptake in the fruit. Furthermore, biostimulant application positively affected the physicochemical properties of soil. The results obtained confirm that the application of biostimulants can be suitable for improving crop sustainability and adaptability under conditions of water stress in semi-arid field regions.

## 1. Introduction

Agriculture in the Mediterranean region is affected by climate change, which is characterized by the recurrence of extreme weather events (heat waves, droughts), the impact of which could be increasingly negative in the near future [1,2]. In these regions, the lack of water and the scarcity of arable soils directly affect crops and are among the main problems facing them [3,4,5]. Over the last decade, the Mediterranean has had some of the most severe droughts on record [6]. Indeed, research show that the frequency and severity of droughts have increased since the early 2000s, putting significant strain on water supplies and crop production [6,7]. Climate research indicate that these tendencies will intensify, with rising temperatures and decreased precipitation [8]. These changes provide a significant challenge to the region’s agricultural sustainability, necessitating the adoption of agricultural technologies that are tailored to these changing conditions [9].

Tomatoes (i.e., *Solanum lycopersicum* L.; “Campbell 33 cultivar”) are considered one of the world’s most consumable and economically attractive crops due to their high yield and short shelf life, particularly in the Mediterranean region, including Morocco [10,11]. These plants are one of the most widely consumed both globally and locally. For instance, Morocco produces the equivalent of 3 million tons of tomatoes, most of which are destined for fresh consumption [11]. Moreover, tomato fruit plays an important role in protecting human health, thanks to its richness in minerals, vitamins, essential amino acids, lycopene, ascorbic acid, antioxidants, and dietary fiber [10,12,13]. These compounds are essential in reducing oxidative stress and inflammation, which supports the immune system and helps to protect against chronic diseases such as diabetes and various cancers [13]. Despite these benefits, tomato plants are very sensitive to water stress, which greatly affects their productivity and fruit quality [10,14].

Currently, sustainable agricultural strategies have been developed to mitigate the adverse effects of drought stress on plants [15,16]. Among the mitigation strategies applied are the application of biostimulants involving arbuscular mycorrhizal fungi (AMF) and organic fertilizers including vermicompost (C) to the soil, which are thought to play a positive role in mitigating various biotic and abiotic stresses, including water scarcity [17].

AMF have emerged as important biostimulants in enhancing plant resilience, particularly in the face of droughts and other environmental challenges [18]. These helpful microorganisms develop symbiotic relationships with a diverse variety of different horticultural crops, e.g., *Ocimum basilicum*, *Lactuca sativa*, *Punica granatum*, and *S. lycopersicum* [19]. AMF regulate plant growth and development by forming extra-radical mycelium around the roots, which are inaccessible to roots or even root hairs, enabling the interchange of nutrients and water for carbon sources such as sugars and lipids [20,21]. Plants inoculated with AMF, such as *Festuca ovina*, *Opuntia ficus-indica*, and *Zea mays*, enhance plant growth and improve the absorption of nutrients, especially nitrogen (N) and phosphorus (P), demonstrating the usefulness of AMF to various plant species [22,23,24]. Recent research suggests that mycorrhizal fungi do more than just aid nutrient uptake. They also play a major role in stimulating microbial and enzymatic activity, enhancing photosynthesis, and increasing water uptake and flexibility in the face of abiotic and biotic stresses, ultimately leading to increased yields [24,25,26,27].

The application of organic fertilizers, of which C is one, plays several roles in supporting different horticultural crops under drought conditions [28,29,30]. Vermicompost is recognized as an essential soil amendment that enhances resistance and tolerance to abiotic stresses across various plant species [28,31]. Further, it has also been reported that this organic fertilizer significantly influences plant cultivation by improving the physical, chemical, and biological characteristics of dry soils [32,33]. Previous studies have shown that C can stimulate plant nutrient uptake and increase mineral nutrition [34,35]. For instance, in a study conducted by Paymaneh et al. [32], it was found that applying boosted P and zinc increased absorption by 30 and 35%, respectively, in pistachio seedlings. Furthermore, C plays a pivotal role in enhancing enzymatic antioxidants including superoxide dismutase, catalase, peroxidase and polyphenol oxidase, and stomatal conductance, and in increasing rates of photosynthesis and transpiration and phytohormone signaling including auxins, cytokinins, gibberellins, ethylene, and abscisic acid; it also regulates reactive oxygen species (ROS) [36,37,38]. It helps improve crop quality, productivity, and yield under drought conditions [28,39].

On the other hand, several previous studies have shown that the use of AMF+C is considered a biostimulant [17]. It helps to improve the growth and physiological and biochemical capabilities of plants, such as cacti [37] and canola [30], under water stress. These include increasing the plants’ ability to absorb water and nutrients, enhancing root growth, stimulating metabolism and growth, and improving immunity to abiotic stresses [37,40].

Thus far, we lack data on the impact of applying C either alone or in conjunction with AMF on the quality of tomato fruits under water stress conditions in the Mediterranean region of Morocco in particular. Furthermore, few studies have been carried out to test the appropriate ratio of AMF-assisted application of indigenous animal C to improve plants yield, as well as fruit quality, improving soil quality in semi-arid regions [41]. This study aimed to evaluate the reciprocal influences between applied biostimulants/organic fertilizers, highlighting the importance of improving tomato fruit production and quality under field drought conditions. The objectives of this research examined (1) the effect of different ratios of C produced from horse manure alone, or in the presence of AMF, on the growth performance and production of tomato plants, with particular emphasis on understanding how these vital components interact under drought conditions; (2) identifying the mechanism of a succession of these biostimulants/organic fertilizers in improving tomato fruit quality under drought conditions; (3) identifying which of the applied percentages of C is most appropriate with or without AMF to improve soil and tomato fruit quality under water stress conditions; (4) providing a suitable biostimulant formula for tomato plants, helping to improve their ability to adapt to difficult environmental conditions and achieve sustainable, high-quality production in the face of challenges.

## 2. Results

### 2.1. Yield, Fruit Growth, and Root Colonization Changes

Data in Table 1 illustrate a significant improvement in fruit yield and growth, including fruit shape index (FSI), fruit fresh weight (FW), and fruit dry weight (DW) for tomato fruits planted under well-watered (WW) conditions by all treatments applied. On the DS (drought-stressed) condition, a marked reduction in yield and fruit growth was observed compared with WW conditions; however, on DS conditions, the application of AMF, C1, C2, AMF+C1, and AMF+C2 improved yield (43, 63, 67, 60, and 50%, respectively) and DW (23, 44, 50, 48 and 49%, respectively), compared with control plants.

Compared with WW plants, the frequency (F) and intensity (I) of AMF colonization were significantly reduced under DW in tomato plants; however, under DS conditions, AMF, AMF+C1, and AMF+C2 treatments resulted in the significant stimulation of F and I mycorrhization by 130, 117, and 91%, respectively, compared with the non-inoculated DS control (Table 1).

### 2.2. Osmotic Solutes Changes

**Figure 1:**
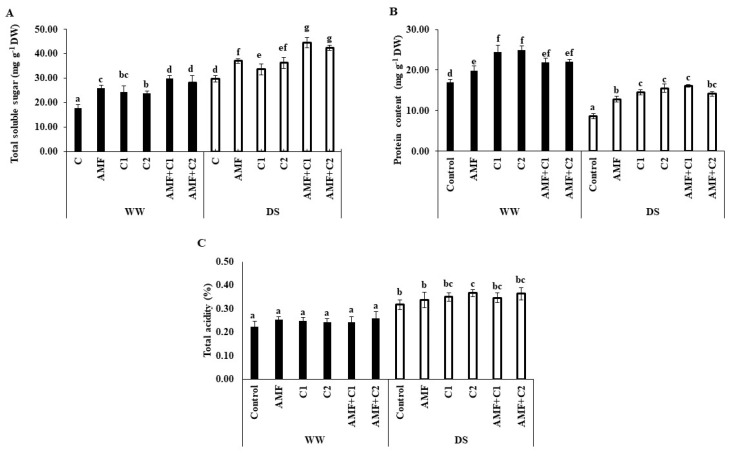
Effect of applied water regimes on (**A**) total soluble sugar, (**B**) protein content, and (**C**) total acidity of tomato, with Control: control treatment; AMF: plants inoculated with AMF consortium; C1: plants amended with 5 t ha^−1^ of organic amendment; C2: plants amended with 10 t ha^−1^ of organic amendment; AMF+C1: plants inoculated with the AMF consortium and amended in C1; AMF+C2: plants inoculated with the AMF consortium and amended in C2. Data are mean ± SE of 3 biological replicates. Means followed by the same letters are not significantly different at *p* < 0.05 (Tukey’s test).

Figure 1 reveals a significant increase in total soluble sugar (TSS) and total acidity (TAC) accumulation in tomato fruits growing under DS conditions; however, protein content was reduced under the same conditions, in comparison with WW watered plants. Furthermore, AMF, C1, C2, AMF+C1, and AMF+C2 plants increased TSS accumulation (24, 13, 22, 49, and 42%, respectively), TAC (6, 11, 16, 9, and 15%, respectively) and protein content (49, 69, 81, 87, and 65%, respectively), compared with control plants tested under DS conditions. In WW conditions, TSS and protein content increased substantially with the application of biostimulants, with a particularly marked increase recorded with AMF+C1 for TSS contents, and C1 for protein.

### 2.3. Carotenoid and Lycopene Content Changes

**Figure 2:**
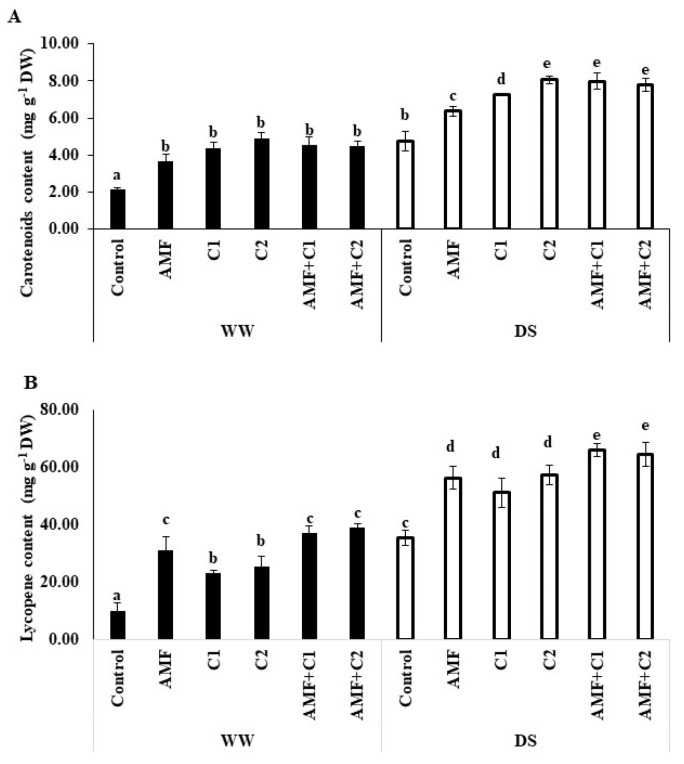
Effect of applied water regimes on (**A**) Carotenoid’s content and (**B**) Lycopene, with Control: control treatment; AMF: plants inoculated with AMF consortium; C1: plants amended with 5 t ha^−1^ of organic amendment; C2: plants amended with 10 t ha^−1^ of organic amendment; AMF+C1: plants inoculated with the AMF consortium and amended in C1; AMF+C2: plants inoculated with the AMF consortium and amended in C2. Data are mean ± SE of 3 biological replicates. Means followed by the same letters are not significantly different at *p* < 0.05 (Tukey’s test).

Figure 2 illustrates the significant effects of drought and the application of biostimulant/organic fertilizers on carotenoid and lycopene content in tomato fruits. Under WW conditions, the application of both AMF and/or C positively influenced these pigments, when compared to the WW control plants. Under DS, an increase in both carotenoid and lycopene levels was observed in the tomato fruit, surpassing the levels in WW plants. Additionally, biostimulant application under DS led to a further increase in the accumulation of these pigments. The recorded increases in lycopene and carotenoid content were, respectively, 34 and 59% with AMF treatment, 53 and 45% for C1-treated plants, 69 and 87% for C2 treatment, 69 and 87% for AMF+C1 treatment, and 64 and 82% for AMF+C2 treatment, compared with control plants under DS conditions.

### 2.4. Ascorbic Acid and Free Amino Acids Changes

**Figure 3:**
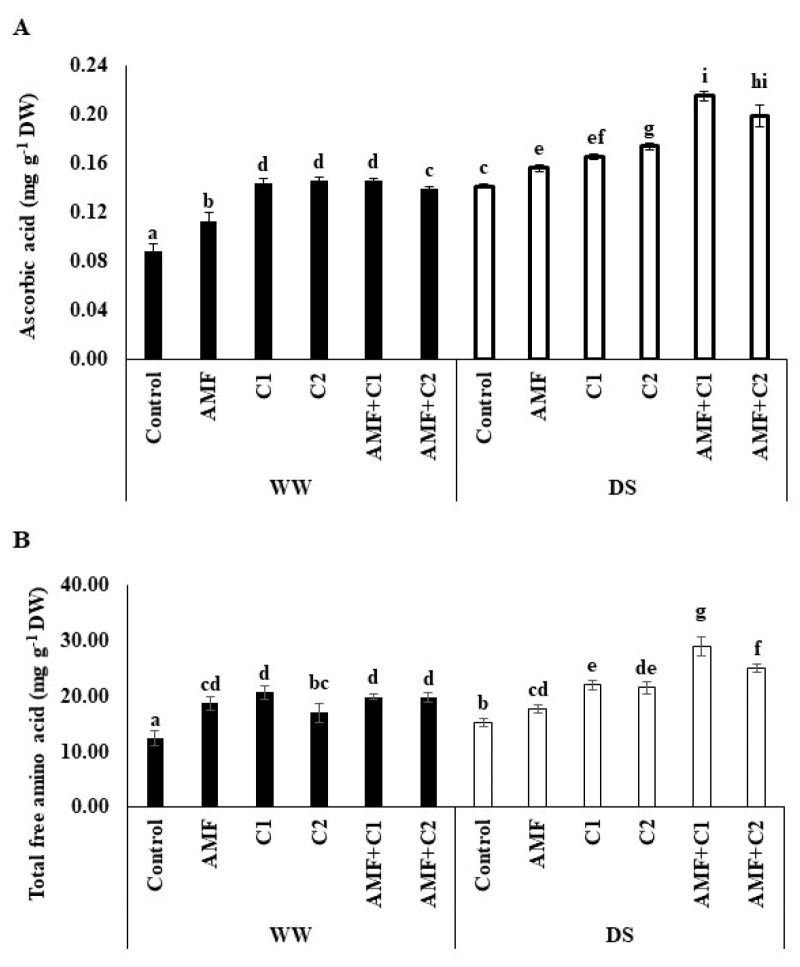
Effect of applied water regimes on (**A**) Ascorbic acid and (**B**) Total free amino acid, with Control: control treatment; AMF: plants inoculated with AMF consortium; C1: plants amended with 5 t ha^−1^ of organic amendment; C2: plants amended with 10 t ha^−1^ of organic amendment; AMF+C1: plants inoculated with the AMF consortium and amended in C1; AMF+C2: plants inoculated with the AMF consortium and amended in C2. Data are mean ± SE of 3 biological replicates. Means followed by the same letters are not significantly different at *p* < 0.05 (Tukey’s test).

Results illustrated in Figure 3 illustrate the high accumulation of ascorbic acid and the content of free amino acids in tomato fruit subjected to DS from treated and non-treated plants compared to WW plants. In addition, the application of AMF and/or C1 and C2 revealed a greater accumulation of these compounds under DS conditions. The most pronounced increases in ascorbic acid and total free amino acid were observed in the AMF+C1 treatment under DS, by 52 and 90%, respectively, compared to the DS control.

### 2.5. Phenol, Flavonoid, and DPPH Activity Changes

**Figure 4:**
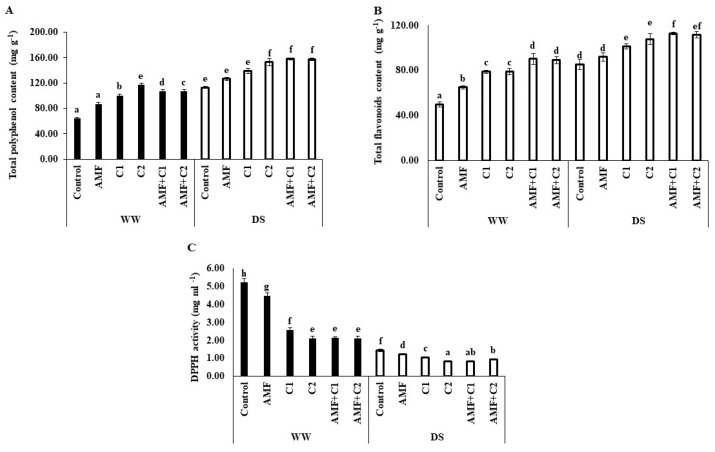
Effect of applied water regimes on (**A**) Total phenolic content, (**B**) Total flavonoids content, and (**C**) DPPH activity, with Control: control treatment; AMF: plants inoculated with AMF consortium; C1: plants amended with 5 t ha^−1^ of organic amendment; C2: plants amended with 10 t ha^−1^ of organic amendment; AMF+C1: plants inoculated with the AMF consortium and amended in C1; AMF+C2: plants inoculated with the AMF consortium and amended in C2. Data are mean ± SE of 3 biological replicates. Means followed by the same letters are not significantly different at *p* < 0.05 (Tukey’s test).

Drought stress significantly affected the levels of bioactive compounds in the plants, altering total phenolic content (TPC), total flavonoid content (TFC), and 2,2-diphenyl-1-picrylhydrazyl (DPPH) activity specifically, as shown in Figure 4. Under DS conditions, there was a significant increase in (TPC) and TFC accumulation, and improved ability to scavenge DPPH free radicals was also demonstrated, compared to plants under WW conditions. In addition, the application of biostimulants/organic fertilizers under DS conditions led to a significant increase in TPC and TFC, with increases of, respectively, 12 and 18%, 23 and 19%, 35 and 26%, 40 and 32%, and 39 and 31%, observed in plants treated with AMF, C1, C2, AMF+C1, and AMF+C2, respectively, compared with control plants under the same drought conditions.

In addition, these treatments under DS conditions also demonstrated a more effective ability to neutralize DPPH free radicals, indicated by a decrease in IC_50_ values of 5, 28, 43, 43, and 34%, respectively, compared to control plants. Furthermore, AMF and/or C application significantly improved TFC, TFC, and DPPH activity under WW conditions, compared with control plants under the same conditions.

### 2.6. Plant Nutrient Uptake Changes

The nutrient content of P, N, and potassium (K) in tomato fruit, in response to drought stress and biostimulant applications, is presented in Table 2. The results show that DS plants showed a decrease in plant nutrient uptake compared with WW plants. In addition, the application of biostimulants improved mineral nutrient content under drought stress. Yet the most significant improvement was observed in plants treated with AMF+C with 140, 23, and 74% improvement, respectively, for P, N, and K.

### 2.7. Soil Analysis

Table 3 data suggest that soil characteristics were affected by DS conditions compared with WW conditions. Under WW conditions, however, the application of biostimulants/organic fertilizers showed a significant increase in soil parameters compared with WW controls. Furthermore, as compared with the DS control, the presented results illustrate that amending the soil under DS conditions resulted in a significant increase in total organic carbon (TOC), available phosphorus (AP), and N levels as a response to biostimulants treatments.

### 2.8. Thermal Mapping of Growth and Biochemical Characteristics of Tomato Fruit Quality

**Figure 5:**
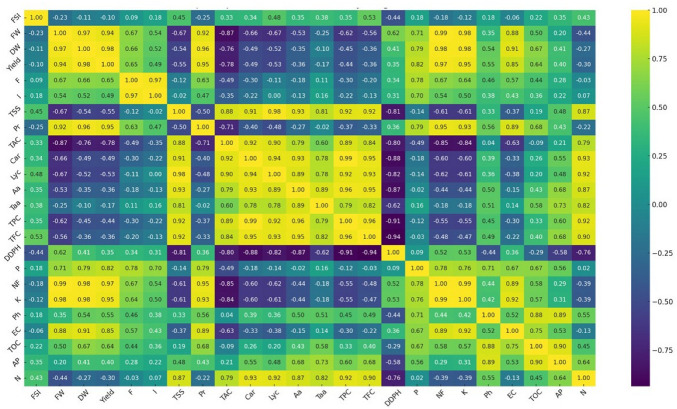
Heat map analyses of tomato fruit subjected to different treatments, with FSI: fruit shape index; FW: fresh fruit weight; DW: fresh dry weight; F: frequency of mycorrhization; I: intensity of mycorrhization; TSS F: total soluble sugar in fruit; Pr: protein content; TAC: total acidity; Car: carotenoids content; Lyc: lycopene; Aa: ascorbic acid content; Faa: free amino acids; TPC: total polyphenol content; TFC: total flavonoid content; DDPH: scavenging capacity of 2,2-diphenyl-1-picrylhydrazyl; P: phosphorus content in fruit; Nf: nitrogen content in fruit; K: potassium content in fruit; EC: electrical conductivity; TOC: total organic carbon; AP: available phosphorus in soil; N: nitrogen content in soil.

The heatmap presented in Figure 5 depicts the interrelationships between several growth and biochemical characteristics of tomato fruit treated with different treatments using a color gradient ranging from yellow (high positive correlation) to purple (strong negative correlation). There are clear patterns of association among the data points; for example, the positive correlation between yield and key nutrients such as N, P, and K and soil nutrients emphasizes their relevance in fruit production and development. Another strong positive correlation was observed linking TSS and fruit-free amino acids. In addition, carotenoids and lycopene have a significant beneficial association. The heatmap also shows that ascorbic acid has a modest association with amino acids. Conversely, TPC and TFC exhibit a high negative connection with DPPH radical scavenging ability.

## 3. Discussion

Changes in yield, including fruit quality, and the biochemical characteristics of tomato yield were studied with the application of an AMF consortium with and without two rates of organic fertilizers. The plants were grown in open fields, under water-stressed conditions in a semi-arid environment. Water-stressed conditions are generally found to adversely affect crop productivity and quality, either through water scarcity or by impairing the availability and assimilation of mineral nutrients.

Mycorrhizal symbiosis is a key factor in plants’ ability to withstand drought conditions [15]. Our results revealed that DS reduced the rate of mycorrhization. These findings are consistent with previous research, which found that the colonization potential of AMF-inoculated plant roots reduced when grown in water-limited soils [29,42]. This suggests that DS damages mycorrhizal structures by limiting spore germination or by decreasing normal symbiosis [43,44]. Furthermore, the combination of AMF and C1 and C2 decreased root infection under DS. This may be due to the P content of C, which reduces the plants’ need for AMF colonization [45,46]. Furthermore, composted organic waste may include breakdown products that hinder mycorrhizal fungi, as AMF development is heavily impacted by soil conditions and management approaches [45].

For the current study, drought led to a significant reduction in yield and fruit growth parameters. Drought stress results in the growth inhibition of tomato plants by decreasing their stems’ cross-sectional areas and the diameter of xylem vessels which, in turn, affects plant water transport efficiency and fruit water content [47,48]. Furthermore, drought stress also affects specific and hydraulic conductivity in tomato plants, resulting in reduced plant growth and fruit fresh weight [49]. This results is consistent with several previous studies, e.g., research carried out on lettuce by Ouhaddo et al. [42] and on tomatoes by Lahbouki et al. [10]. Interestingly, AMF and C, when applied at different rates to tomato plants, can have varying positive effects on yield and fruit quality in drought conditions.

The beneficial effects of AMF on increasing the size and weight of tomato fruit, as well as increasing the yield under water-deficit conditions, can be attributed to their ability to enhance the uptake of available water in the root zone through the soil–plant continuum [50]. This is crucial for maintaining turgor pressure and metabolic activities in developing fruits, resulting in improved growth outcomes [51,52]. Furthermore, fungi contribute to the absorption and facilitation of nutrient uptake, particularly by enhancing the uptake of P in plants [24]. Phosphorus is vital for cellular energy transfer, photosynthesis, and the synthesis of nucleic acids, all of which contribute to increased fruit size and biomass [53]. In addition to P, AMF aids in the absorption of other essential nutrients such as N and K [54,55]. These elements play key roles in the fruiting process of plants, leading to increased yields [53]. On the other hand, C significantly enhances the productivity of tomato plants by supplying essential nutrients such as P, N, and K in forms readily accessible to the plants. Moreover, the C improves soil aeration and aggregation, thereby boosting the soil’s capacity to hold water [56,57]. This improvement provides plants with more consistent access to water, positively affecting soil structure. Additionally, C is rich in humic substances and hormonal activity, which stimulates plant growth which, in turn, stimulates increased cell division and expansion, contributing to the growth and development of plant tissue and thus increasing fruit size and yield [28,58].

Furthermore, our results also demonstrated that the dual application of AMF and C1 significantly increased plant growth parameters. The synergy between AMF and a moderate amount of C might create an optimal balance of nutrients available to the tomato plants. AMF enhances the uptake of P and other nutrients, which, when combined with the balanced nutrient profile provided by the lower rate of C, could lead to more efficient use of nutrients, promoting healthier plant growth and fruit development [59,60].

Tomato fruit quality is strongly affected by drought stress, mainly due to changes in metabolic pathways that influence the accumulation of TSS, TAC, and secondary metabolites. In this study, an increase in TSS, TAC, and free amino acids was observed in tomato fruit grown under DS. The accumulation of these compounds in plant tissues could also be linked to the activation of starch hydrolysis during water stress, which acts as an osmotic adjustment mechanism to keep cell turgor pressure stable and to support overall physiological characteristics [42,61,62]. In addition, the response of plant fruits to drought-induced oxidative stress is characterized by a significant modulation of oxidative activities.

In this study, enhanced levels of carotenoids, lycopene, TAC, and ascorbic acid indicate an enhanced antioxidant defense system aimed at neutralizing the ROS generated under stress conditions [62,63]. Carotenoids and lycopene, known for their bright pigmentation in tomatoes, play an important role beyond aesthetic appearance [61]. Previous studies have shown that these compounds have strong antioxidant properties, effectively quenching singlet oxygen and various other volatile oxygen species, thus preventing potential damage to cellular components caused by water stress [24,63,64].

In the presence of AMF and/or C, under drought stress, the concentrations of these pigments increased. Several previous studies have shown that the application of AMF/C increases carotenoid and lycopene levels [32,65]. This may be due to the ability of mycorrhizae to increase P uptake, which ensures the availability of ATP required for the carotenoid biosynthesis pathway, ultimately leading to lycopene production [66,67]. On the other hand, mycorrhizae modulate phytohormones, in particular abscisic acid, and can affect lycopene biosynthesis. Furthermore, C aids in the formation of acetyl-CoA and influences the activity of pyruvate kinase and phosphofructokinase due to its richness in nutrients, especially potassium, which plays an important role in the synthesis of lycopene [68]. In addition, the rise in ascorbic acid levels can be attributed to its crucial function as an antioxidant within the plant’s defense mechanism against oxidative stress. Due to its ability to directly scavenge various types of harmful oxidants, C protects plant cells from oxidative damage [24,69].

Similarly, the increased observation of TFC and TPC contents supports the tomato fruit’s superior ability to defend against oxidative stress. They work by giving ROS electrons, which neutralize them and stop oxidative damage [70]. Additionally, these substances function as metal chelators and aid in the stability of cell membranes, strengthening their ability to fend off oxidative stress due to drought [71,72]. The positive effects of AMF on the expression of genes related to phenylpropanoids, flavonoids, and isoflavonoids may be linked to the accumulation of this compound. As previously observed, AMF could modify plant phytohormones such as cytokinins and auxin during drought stress, potentially increasing the production of phenolic compounds [16,73]; however, the effect of C on flavonoids and other phenolic acids is unclear. A possible explanation for the increase in these compounds is that organic amendment contains high levels of humic and fulvic acids, which have been associated with the production of phenolic metabolites [74].

Plants facing drought conditions and growing in an environment enriched with biostimulants/ organic fertilizers demonstrated an enhanced ability to cope with stress by activating antioxidants better than other plants. As shown in several studies, the accumulation of phenolic compounds is directly linked to improved DPPH activity in IC_50_ plants [75,76]. This suggests that the increased accumulation of these compounds may be a contributing factor in improving the plant’s ability to regulate and suppress oxidative activities that can be harmful during periods of stress such as drought.

## 4. Materials and Methods

### 4.1. Plant and Biostimulants/Organic Fertilizers Materials

To explore the effects of biostimulants on water stress and tomato fruit quality, a field study was conducted on *S. lycopersicum* L., or “Campbell 33 cultivar,” which were obtained from the National Institute of Agricultural Research (Marrakech, Morocco). Seeds were initially sterilized in 10% sodium hypochlorite for 10 min, then placed on 1% agar plates with half-strength MS medium to check microbial contamination. These plates were incubated in the dark at 28 °C for five days. Subsequently, seedlings were moved to sterilized plastic trays filled with peat vegetable; after 15 days, seedlings exhibiting uniform growth were transferred to the field for planting.

The mycorrhizal inoculum utilized in this study contained spores, mycelium, and infested maize root fragments. It consisted of 1034 spores per 100 g soil in 22 species [75].

The organic fertilizer used in this study was a C containing a mixture of horse manure and straw, enriched with *Eisenia fetida* earthworms. The C is produced locally, as described by [41]. Physical and chemical properties included a pH of 7.7, an electrical conductivity of 4.89 mS cm^−1^, an organic matter concentration of 438.8 mg g^−1^, available phosphorus of 0.29 mg g^−1^, a total organic carbon content of 280 mg g^−1^, and total nitrogen measured at 6.2 mg g^−1^.

AMF and C were applied during the planting phase, added directly to the soil and distributed evenly throughout the plots. This process was conducted before planting the tomato seedlings to ensure optimal interaction between the AMF and plant roots, and to optimize soil fertility with C.

### 4.2. Field Experimental Design Plant Assay

The field experiment was conducted at a private agricultural field in the SAADA district of Marrakesh, Morocco, (31°37′39.9″ N and 08°07′46.7″ W). The region experiences a semi-arid, typically Mediterranean climate, with an average annual temperature of 19.6 °C, ranging from an average monthly temperature of 7 °C in January to 30 °C in July. The average annual rainfall is 250 mm, and most of it occurs in the period from February to June [77]. The agricultural plot is equipped with a drip irrigation system, and there is no record of employing pesticides or chemical fertilizers in past cultivation cycles.

The soil in the field has a sandy clay loam texture, comprising 52% sand, 24% clay, and 24% silt. The electrical conductivity (EC) of the irrigation water measures 3.02 ± 0.25 dS m^−1^, while the pH level is 7.22 ± 0.32.

The experimental setup was organized in a randomized block design, dividing the field into 36 plots (0.8 m wide and 0.5 m apart) with 6 rows each, hosting about 8 plants per plot. Each plot was randomly allocated to two watering regimes (well-watered or water-stressed) and biostimulants/C treatments, as detailed in Table 4 and Figure S1, with three replications per treatment, totaling 36 plots (2 × 6 × 3).

Well-watered (WW) plants were irrigated using drip emitters providing 8 L per hour, while drought-stressed (DS) plants received water at 4 L per hour from similar emitters. Irrigation for both groups was scheduled twice daily in two 30 min intervals, one in the morning and the other before sunset. This irrigation regime was applied consistently 5 days a week, from the first week of planting until harvest.

### 4.3. Mycorrhization Assessments

Colonization by AMF was determined using the Trypan blue staining method [78] and observed under a Zeiss Axioskop 40 microscope at 40–100× magnification. Briefly, 1 g of roots (fresh weight) was cleaned with 10% KOH at 90 °C for 1 h, washed with distilled water, and acidified in 5% HCl. A 5% Trypan blue solution was used to stain fixed root samples. Fa and I of AMF infection were determined using the equations below as described by Trouvelot and Kough [79]:

$$\mathrm{AMF}\;\mathrm{infection}\;\mathrm{frequency}=\frac{\mathrm{Infected}\;\mathrm{root}\;\mathrm{segments}}{\mathrm{Total}\;\mathrm{root}\;\mathrm{segments}}\times 100$$

$$\mathrm{AMF}\;\mathrm{infection}\;\mathrm{intensity}=\frac{\left(95n5+70n4+30n3+5n2+\;n1\right)}{\mathrm{Total}\;\mathrm{root}\;\mathrm{segments}}$$

where n5 corresponds to the number of roots with an infection level of 5 (infection rate 90–100%); n4 corresponds to the number of roots at level 4 (infection rate 50–90%); n3 corresponds to the number of roots at level 3 (infection rate at 10–50%); n2 corresponds to the number of roots at infection level 2 (infection rate 1–10%); and n1 corresponds to the number of roots at level 1 (infection rate 0–1%).

### 4.4. Fruit Yield and Growth Measurement

Using a digital caliper, the transverse diameter (TD) and longitudinal diameter (LD) of each tomato fruit were measured, and FSI was calculated as reported by [80] as follows:

$$\mathrm{FSI}=\frac{\mathrm{LD}}{\mathrm{TD}}$$

Tomato FW was measured using an analytical balance (accurate to 0.01 g). The fruit was placed in a drying oven, baked at 105 °C for 1 h, and dried at 80 °C to constant weight, which was recorded as DW. Fruit yield was taken at harvest and expressed as the number of fruits per plant.

### 4.5. Total Soluble Sugar and Protein Content Determination in Fruit Tomatoes

The content of TSS was determined spectrophotometrically by recording the absorbance at 585 nm according to [81]. Finely ground samples of fruit (0.1 g) were homogenized in 4 mL of ethanol (80%). Of the supernatant obtained, 0.2 mL was mixed with 0.2 mL of 5% phenol and 1 mL of concentrated sulfuric acid, then calculated using the standard glucose curve.

The measurement of protein content was started by homogenizing 0.1 g of freeze-dried fruit in a cold mortar with 4 mL of 1 M phosphate buffer (pH 7), supplemented with 5% polyvinylpolypyrrolidone. After centrifuging the homogenate at 18,000× *g* for 15 min at 4 °C, the content of soluble protein was measured using the Bradford method [82].

### 4.6. Total Acidity Determination in Fruits Tomatoes

Titrimetry was used to determine total acidity (TAC) using a 0.1 N NaOH solution. In a mortar, 1 g of fruit pulp was combined with 20 mL of distilled water. In the presence of phenolphthalein, the juice was filtered and titrated with a 0.1 N NaOH solution [83]. Using the mathematical relationship, total acidity was calculated as follows:

$$\mathrm{TTA}\;\left(\%\right)=\frac{\left(\mathrm{NaOH}\;\mathrm{used}\;\mathrm{volume}\right)\times \left(\mathrm{NaOH}\;\mathrm{molarity}\right)\times \left(\mathrm{milliequivalent}\;\mathrm{factor}\right)}{\mathrm{Sample}\;\mathrm{volume}}\times 100$$

with milliequivalent factor = 0.0064 (citric acid).

### 4.7. Carotenoid and Lycopene Content Determination in Tomato Fruit

Carotenoid concentrations were measured spectrophotometrically at 480, 663, and 645 nm, as described by Zhou et al. [84]. Acetone (80%) was used to extract carotenoids from tomato fruit samples. Their concentrations were determined using the following formula:

$$\mathrm{Carotenoïds}\;\left(\mathrm{mg}/\mathrm{gdw}\right)=\frac{\left[\left(A480+0.114\right)\times \left(A663-0.638\right)\times \left(A645\right)\right]\times V}{1000\times \mathrm{DW}}$$

where, A = absorbance; V = extract final volume, and DW = dry weight.

The Lycopene content of the tomato fruit was quantified according to the methodology described by Roldán-Gutiérrez and Luque de Castro, i.e., spectrophotometrically at 595 nm using an ultraviolet-1800 spectrophotometer (Shimadzu, Kyoto, Japan) [85]. Lycopene concentration was determined by applying an extinction coefficient of ε: 3450.

### 4.8. Ascorbic Acid and Free Amino Acids Content Determination in Tomato Fruit

Ascorbic acid content was determined according to the method described by Adrian and Peiró [86]. The determination of ascorbic acid was determined on the comparison between the volume of 2,6-DCPIP used in this titration and the volume used with a standard ascorbic acid solution at a concentration of 0.1%.

The content of total free amino acids was assessed spectrophotometrically at 760, employing the ninhydrin-assay-based protocol established by Lee and Takahashi, with glycine serving as the calibration standard [87].

### 4.9. Assessment of Phenol and Flavonoid Content in Tomato Fruit

Total polyphenol content, TPC, and DPPH activity were measured on tomato fruit powder obtained after drying at 50 °C for 48 h, then the samples were ground and sieved.

Total polyphenol content was calculated using the Folin–Ciocalteu method [88]. Dried tomato fruit powder was ground in 50 mL of 80% methanol and filtered using a Buchner funnel and Whatman filter paper. The absorbance at 725 nm was determined, with gallic acid as the reference phenolic component.

Total flavonoid content was measured spectrophotometrically at 510, using the method outlined by Tohidi et al. [89]. Dried tomato fruit powder was ground in 50 mL of methanol (80%) and mixed with a 30 mL 5% NaNO_2_ solution and 60 mL of 10% aluminum chloride. The mixture was stopped by spiking 2 mL of 1 M sodium hydroxide. TFC was expressed as mg quercetin per g dry weight.

### 4.10. 2,2-Diphenyl-1-picrylhydrazyl Radical Scavenging Activity

The determination of the scavenging capacity DPPH^·^ of the methanol tomato extract was measured at 517 nm according to the method outlined by Aruwa et al. [90]. The reaction mixture consisted of 50 µL of the tomato extract, added to 2 mL of a 60 mM methanolic solution of DPPH. The group blank was considered to replace the sample with distilled water.

### 4.11. Plant Nutrient Uptake Analysis

The mineral nutrients (N, P, and K) of the tomato fruit were measured. After the samples were oven-dried at 50 °C for 48 h, they were finely crushed, and 1 g was added to each digestion tube along with H_2_SO_4_ for digestion [91,92]. The plant filtrate was analyzed for nitrogen using the Kjeldhal technique, and the amounts of phosphorus were determined using the spectrophotometric method. Fruit K concentration was measured by flame photometry [93].

### 4.12. Soil Analysis

Soil physicochemical parameters were examined after the experiment. Soil samples were taken from around the root zone, air-dried, and then sieved. The pH and EC of a water-based solution were measured as part of the examination. In addition, total organic carbon was assessed using the technique proposed by Aubert [47,94]. Available phosphorus was determined using the approach taken by Olsen and Sommers [95].

### 4.13. Statistical Analysis

Statistical analysis was performed using SPSS 23 software on the average values of three replicates ± standard error (SE). Using ANOVA, followed by a separate Tukey’s test with significance set at *p* < 0.05, the effects of drought, AMF, C levels, and their interactions were assessed (SPSS 23, IBM, Armonk, NY, USA). The heat map characteristics of tomato plants measured under various treatments were plotted using GraphPad^®^ Prism v9.0 software.

## 5. Conclusions

In this study, it was clearly demonstrated that the productivity and quality of tomato fruit are negatively affected by water stress under field conditions. The results indicated that the use of biostimulants/organic fertilizers contributed to improving and increasing tomato quality under drought conditions. The application of AMF and C, either separately or in combination, significantly improved the productivity and growth of tomato fruit, especially in terms of FSI and DW under DS. Additionally, biostimulants/organic fertilizers demonstrated a positive effect in enhancing the accumulation of osmotic substances and improving the performance of antioxidant enzyme systems and bioactive compounds, as well as increasing nutrient uptake (N, P, and K), which contributed to reducing drought-induced oxidative stress. Furthermore, AMF and/or C led to significant improvements in soil fertility, leading to increased organic matter content and nutrient levels, which enhanced the ability of the soil to support plant growth. Based on our findings, we recommend the use of AMF+C1 as a sustainable strategy to increase tomato productivity and improve tomato quality in arid and semi-arid agricultural environments. As promising as these results are, there is still a need for further research to understand the molecular mechanisms underlying these biostimulants in improving tomato resistance to water stress. Further research is also needed that applies these biostimulants to different growing environments and climates during different seasons in order to confirm their effectiveness in enhancing tomato tolerance to water stress.

## Figures and Tables

**Table 1:** Effectiveness of AMF and/or vermicompost doses on growth and yield parameters of tomatoes and AMF colonization, grown under normal and drought conditions.

		Yield/Plant	TD (cm)	LD (cm)	FSI	FW (g/Plant)	DW (g/Plant)	F %	I%
WW	Control	21.33 ± 1.52 ^e^	4.91 ± 0.52 ^cd^	8.36 ± 0.77 ^f-h^	1.70 ± 0.04 ^e^	644.67 ± 22.32 ^f^	18.35 ± 1.75 ^d^	37.84 ± 9.56 ^bc^	22.43 ± 1.14 ^b^
	AMF	25.33 ± 2.13 ^f^	5.26 ± 0.51 ^ef^	8.69 ± 0.24 ^gh^	1.65 ± 0.07 ^de^	674.67 ± 19.22 ^g^	20.03 ± 1.68 ^e^	72.16 ± 16.36 ^g^	54.11 ± 3.52 ^g^
	C1	31.67 ± 1.54 ^g^	4.95 ± 0.27 ^de^	8.92 ± 0.12 ^h^	1.80 ± 0.03 ^f^	689.67 ± 22.57 ^gh^	22.74 ± 2.09 ^ef^	48.16 ± 8.36 ^de^	26.67 ± 0.96 ^bc^
	C2	38.00 ± 1.36 ^i^	5.03 ± 0.22 ^e^	9.06 ± 0.09 ^h^	1.80 ± 0.03 ^f^	702.00 ± 14.25 ^h^	24.02 ± 1.41 ^f^	47.55 ± 7.25 ^d^	27.82 ± 1.35 ^c^
	AMF+C1	37.00 ± 1.42 ^hi^	6.88 ± 0.44 ^g^	9.27 ± 0.12 ^ij^	1.34 ± 0.05 ^ab^	704.00 ± 11.65 ^h^	23.55 ± 2.23 ^ef^	69.13 ± 15.78 ^g^	45.76 ± 3.66 ^f^
	AMF+C2	34.67 ± 1.26 ^gh^	6.93 ± 0.63 ^g^	9.47 ± 0.17 ^j^	1.36 ± 0.05 ^ab^	703.33 ± 12.47 ^h^	24.89 ± 1.45 ^g^	61.37 ± 9.45 ^fg^	40.85 ± 2.32 ^e^
DS	Control	10.00 ± 1.46 ^a^	2.71 ± 0.52 ^a^	4.02 ± 0.32 ^a^	1.48 ± 0.07 ^c^	416.00 ± 13.66 ^a^	10.28 ± 1.08 ^a^	22.39 ± 6.38 ^a^	14.19 ± 0.68 ^a^
	AMF	14.33 ± 2.06 ^b^	3.43 ± 0.22 ^b^	5.52 ± 0.08 ^b^	1.60 ± 0.03 ^d^	447.67 ± 9.52 ^b^	12.61 ± 0.95 ^b^	51.65 ± 8.28 ^e^	34.19 ± 2.36 ^d^
	C1	16.33 ± 2.36 ^cd^	4.08 ± 0.12 ^bc^	5.85 ± 0.30 ^cd^	1.43 ± 0.03 ^bc^	478.33 ± 25.66 ^c^	14.76 ± 1.31 ^bc^	31.84 ± 17.56 ^b^	20.85 ± 1.27 ^b^
	C2	16.78 ± 1.45 ^d^	4.21 ± 0.02 ^c^	6.03 ± 0.36 ^d^	1.43 ± 0.04 ^bc^	495.11 ± 20.52 ^d^	15.37 ± 0.86 ^c^	34.66 ± 5.42 ^b^	23.62 ± 1.09 ^b^
	AMF+C1	16.00 ± 1.76 ^c^	4.22 ± 0.28 ^c^	6.01 ± 0.15 ^d^	1.42 ± 0.06 ^b^	489.67 ± 16.34 ^cd^	15.19 ± 1.28 ^c^	48.5 ± 8.32 ^de^	33.58 ± 1.55 ^d^
	AMF+C2	15.00 ± 2.43 ^bc^	4.09 ± 0.24 ^bc^	6.55 ± 0.27 ^e^	1.60 ± 0.05 ^d^	511.67 ± 35.75 ^de^	15.30 ± 1.12 ^c^	42.86 ± 8.67 ^cd^	28.71 ± 0.68 ^c^

WW: well-watered; DS: drought-stressed plants; TD: transverse diameter of tomato fruit; LD: longitudinal diameter of tomato fruit; FSI: fruit shape index; FW: tomato fresh fruit weight; DW: tomato dry fruit weight; F: root colonization frequency; I: mycorrhization intensity; Control: control treatment; AMF: plants inoculated with AMF consortium; C1: plants amended with 5 t/ha of organic amendment; C2: plants amended with 10 t/ha of organic amendment; AMF+C1: plants inoculated with AMF consortium and amended C1; AMF+C2: plants inoculated with AMF consortium and amended C2. Data represent the means ± standard error (SE) (n = 3). Means in the same column with different letters indicate significant differences at *p* ≤ 0.05.

**Table 2:** Effect of AMF and/or vermicompost doses on mineral nutrition of tomato fruit and AMF colonization cultivated in normal and drought field conditions.

Water Regime	Treatments	Phosphorus (%)	Nitrogen (%)	Potassium (%)
WW	Control	0.06 ± 0.01 ^c^	1.26 ± 0.16 ^d^	2.77 ± 0.41 ^f^
	AMF	0.12 ± 0.02 ^d^	1.37 ± 0.12 ^ef^	2.68 ± 0.36 ^g^
	C1	0.11 ± 0.01 ^de^	1.44 ± 0.18 ^fg^	3.12 ± 0.49 ^hi^
	C2	0.14 ± 0.03 ^ef^	1.53 ± 0.27 ^h^	3.24 ± 0.52 ^ig^
	AMF+C1	0.16 ± 0.02 ^f^	1.50 ± 0.12 ^h^	3.45 ± 0.29 ^g^
	AMF+C2	0.14 ± 0.03 ^ef^	1.48 ± 0.11 ^gh^	3.36 ± 0.48 ^g^
DS	Control	0.05 ± 0.00 ^a^	0.86 ± 0.19 ^a^	1.07 ± 0.29 ^a^
	AMF	0.08 ± 0.00 ^cd^	0.88 ± 0.22 ^ab^	1.15 ± 0.32 ^b^
	C1	0.05 ± 0.01 ^bc^	0.95 ± 0.19 ^b^	1.44 ± 0.24 ^c^
	C2	0.10 ± 0.02 ^de^	0.99 ± 0.16 ^bc^	1.78 ± 0.16 ^de^
	AMF+C1	0.12 ± 0.01 ^e^	1.06 ± 0.18 ^c^	1.86 ± 0.16 ^e^
	AMF+C2	0.06 ± 0.00 ^c^	1.03 ± 0.11 ^c^	1.75 ± 0.35 ^de^

WW: well-watered; DS: drought-stressed plants; Control: control treatment; AMF: plants inoculated with AMF consortium; C1: plants amended with 5 t/ha of organic amendment; C2: plants amended with 10 t/ha of organic amendment; AMF+C1: plants inoculated with AMF consortium and amended C1; AMF+C2: plants inoculated with AMF consortium and amended C2. Data represent the means ± standard error (SE) (n = 3). Means in the same column with different letters indicate significant differences at *p* ≤ 0.05.

**Table 3:** Effect of AMF and/or vermicompost doses on soil physico-chemical parameters before and after the experiment.

Water Regime	WW						DS					
Treatments	Control	AMF	C1	C2	AMF+C1	AMF+C2	Control	AMF	C1	C2	AMF+C1	AMF+C2
pH	7.74 ± 0.45 ^a^	7.80 ± 0.33 ^ab^	7.84 ± 0.52 ^bc^	7.93 ± 0.82 ^c^	7.86 ± 0.63 ^bc^	7.91 ± 0.28 ^c^	7.74 ± 0.51 ^a^	7.84 ± 0.76 ^bc^	7.81 ± 0.38 ^a–c^	7.86 ± 0.34 ^bc^	7.88 ± 0.28 ^bc^	7.88 ± 0.31 ^bc^
EC (mS cm^−1^)	1.75 ± 0.36 ^cd^	1.72 ± 0.54 ^cd^	1.82 ± 0.41 ^cd^	1.79 ± 0.34 ^cd^	1.83 ± 0.35 ^d^	1.81 ± 0.41 ^cd^	1.52 ± 0.57 ^a^	1.55 ± 0.45 ^ab^	1.67 ± 0.13 ^bc^	1.65 ± 0.28 ^bc^	1.72 ± 0.35 ^cd^	1.72 ± 0.21 ^cd^
TOC (%)	0.92 ± 0.12 ^b^	1.15 ± 0.31 ^c^	1.35 ± 0.28 ^e–g^	1.48 ± 0.19 ^gh^	1.43 ± 0.25 ^fg^	1.52 ± 0.41 ^h^	0.88 ± 0.11 ^a^	0.95 ± 0.08 ^b^	1.25 ± 0.19 ^d^	1.21 ± 0.25 ^d^	1.39 ± 0.14 ^e–g^	1.41 ± 0.32 ^e–g^
AP (mg/kg)	26.74 ± 3.36 ^a^	43.3 ± 4.15 ^c^	84.36 ± 3.86 ^ef^	89.19 ± 4.18 ^fg^	102.75 ± 5.85 ^i^	91.95 ± 6.22 ^gh^	33.84 ± 3.68 ^b^	53.48 ± 5.32 ^c^	72.19 ± 5.85 ^d^	88.32 ± 4.86 ^fg^	99.40 ± 5.85 ^hi^	99.39 ± 5.56 ^hi^
N (g/kg)	0.70 ± 0.09 ^a^	0.98 ± 0.12 ^cd^	0.84 ± 0.09 ^b^	1.05 ± 0.15 ^de^	1.02 ± 0.19 ^de^	1.08 ± 0.11 ^e^	0.85 ± 0.06 ^b^	1.12 ± 0.25 ^e–g^	1.27 ± 0.19 ^fg^	1.30 ± 0.21 ^g^	1.34 ± 0.21 ^g^	1.34 ± 0.14 ^g^

WW: well-watered; DS: drought-stressed plants; EC: electrical conductivity; TOC: total organic carbon; AP: available phosphorus; N: nitrogen; Control: control treatment; AMF: plants inoculated with AMF consortium; C1: plants amended with 5 t/ha of organic amendment; C2: plants amended with 10 t/ha of organic amendment; AMF+C1: plants inoculated with AMF consortium and amended C1; AMF+C2: plants inoculated with AMF consortium and amended C2. Data represent the means ± standard error (SE) (n = 3). Means in the same column with different letters indicate significant differences at *p* ≤ 0.05.

**Table 4:** Different applied treatments in the study.

Treatments	Water Regime	
Control	well-watered	Plants non-inoculated and non-amended
AMF	water-stressed	Plants inoculated with AMF (200 g/plot)
C1		Plants amended with vermicompost at 0.6 kg/plot (5 t ha^−1^)
C2		Plants amended with vermicompost at 1.2 kg/plot (10 t ha^−1^)
AMF+C1		Combined application of AMF and C1
AMF+C2		Combined application of AMF and C2

## Data Availability

Data are contained within the article and Supplementary Materials.

## Supplementary Materials

The following supporting information can be downloaded at: https://www.mdpi.com/article/10.3390/plants13111449/s1, Figure S1: Experimental design map with, Control: control treatment; AMF plots: plants inoculated with AMF consortium; C1 plots: plants amended with 5 t ha^−1^ of organic amendment; C2 plots: plants amended with 10 t ha^−1^ of organic amendment; AMF+C1 plots: plants inoculated with the AMF consortium and amended in C1; AMF+C2 plots: plants inoculated with the AMF consortium and amended in C2.

## References

[1] Abd-Elmabod S.K., Muñoz-Rojas M., Jordán A., Anaya-Romero M., Phillips J.D., Jones L., Zhang Z., Pereira P., Fleskens L., van Der Ploeg M. (2020). Climate Change Impacts on Agricultural Suitability and Yield Reduction in a Mediterranean Region. Geoderma.

[2] Gaaloul N., Eslamian S., Katlance R. (2021). Impacts of Climate Change and Water Resources Management in the Southern Mediterranean Countries. Water Product. J..

[3] El-Sanatawy A.M., El-Kholy A.S.M., Ali M.M.A., Awad M.F., Mansour E. (2021). Maize Seedling Establishment, Grain Yield and Crop Water Productivity Response to Seed Priming and Irrigation Management in a Mediterranean Arid Environment. Agronomy.

[4] Amiri N., Lahlali R., Amiri S., El Jarroudi M., Khebiza M.Y., Messouli M. (2021). Development of an Integrated Model to Assess the Impact of Agricultural Practices and Land Use on Agricultural Production in Morocco under Climate Stress over the next Twenty Years. Sustainability.

[5] Mansour E., Desoky E.-S.M., Ali M.M.A., Abdul-Hamid M.I., Ullah H., Attia A., Datta A. (2021). Identifying Drought-Tolerant Genotypes of Faba Bean and Their Agro-Physiological Responses to Different Water Regimes in an Arid Mediterranean Environment. Agric. Water Manag..

[6] Cook B.I., Anchukaitis K.J., Touchan R., Meko D.M., Cook E.R. (2016). Spatiotemporal Drought Variability in the Mediterranean over the Last 900 Years. J. Geophys. Res. Atmos..

[7] Tramblay Y., Koutroulis A., Samaniego L., Vicente-Serrano S.M., Volaire F., Boone A., Le Page M., Llasat M.C., Albergel C., Burak S. (2020). Challenges for Drought Assessment in the Mediterranean Region under Future Climate Scenarios. Earth-Sci. Rev..

[8] Majid I., Aziane N., Oubbih J., Ramah M., Chakiri S. (2024). Impact of Climate Change on Cultivated Areas and Crop Yields for Cereals and Pulses in the Zaër Region (Morocco). Ecol. Eng. Environ. Technol..

[9] Wakweya R.B. (2023). Challenges and Prospects of Adopting Climate-Smart Agricultural Practices and Technologies: Implications for Food Security. J. Agric. Food Res..

[10] Lahbouki S., Meddich A., Ben-Laouane R., Outzourhit A., Pari L. (2022). Subsurface Water Retention Technology Promotes Drought Stress Tolerance in Field-Grown Tomato. Energies.

[11] Benabderrazik K., Kopainsky B., Tazi L., Joerin J., Six J. (2021). Agricultural Intensification Can No Longer Ignore Water Conservation–A Systemic Modelling Approach to the Case of Tomato Producers in Morocco. Agric. Water Manag..

[12] Asiamah E., Arthur W., Kyei-Barfour V., Sarpong F., Ketemepi H.K. (2023). Enhancing the Functional and Physicochemical Properties of Tomato (*Solanum lycopersicum* L.) Fruit through Polysaccharides Edible Dipping Technique Coating under Various Storage Conditions. Bioact. Carbohydrates Diet. Fibre.

[13] Yong K.T., Yong P.H., Ng Z.X. (2023). Tomato and Human Health: A Perspective from Post-harvest Processing, Nutrient Bio-accessibility, and Pharmacological Interaction. Food Front..

[14] Chakma R., Saekong P., Biswas A., Ullah H., Datta A. (2021). Growth, Fruit Yield, Quality, and Water Productivity of Grape Tomato as Affected by Seed Priming and Soil Application of Silicon under Drought Stress. Agric. Water Manag..

[15] Nacoon S., Ekprasert J., Riddech N., Mongkolthanaruk W., Jogloy S., Vorasoot N., Cooper J., Boonlue S. (2021). Growth Enhancement of Sunchoke by Arbuscular Mycorrhizal Fungi under Drought Condition. Rhizosphere.

[16] Bahadur A., Batool A., Nasir F., Jiang S., Mingsen Q., Zhang Q., Pan J., Liu Y., Feng H. (2019). Mechanistic Insights into Arbuscular Mycorrhizal Fungi-Mediated Drought Stress Tolerance in Plants. Int. J. Mol. Sci..

[17] Meddich A. (2023). Biostimulants for Resilient Agriculture—Improving Plant Tolerance to Abiotic Stress: A Concise Review. Gesunde Pflanz..

[18] Tian H., Jia Z., Liu W., Wei X., Wang H., Bao G., Li J., Zhou Q. (2023). Effects of Arbuscular Mycorrhizal Fungi on Growth and Nutrient Accumulation of Oat under Drought Conditions. Agronomy.

[19] Mitra D., Dam P., Mondal R., Mahakur B., Al-Tawaha A.R.M., Sangeetha J., Thangadurai D., Chippalakatti P. (2023). Application of Arbuscular Mycorrhiza Fungi in Agricultural and Horticultural Crops. Mycorrhizal Technology.

[20] Trinchera A., Warren Raffa D. (2023). Weeds: An Insidious Enemy or a Tool to Boost Mycorrhization in Cropping Systems?. Microorganisms.

[21] Wang M., Wang Z., Guo M., Qu L., Biere A. (2023). Effects of Arbuscular Mycorrhizal Fungi on Plant Growth and Herbivore Infestation Depend on Availability of Soil Water and Nutrients. Front. Plant Sci..

[22] Klink S., Giesemann P., Hubmann T., Pausch J. (2020). Stable C and N Isotope Natural Abundances of Intraradical Hyphae of Arbuscular Mycorrhizal Fungi. Mycorrhiza.

[23] Tatewaki Y., Higo M., Isobe K. (2023). Impacts of Tillage Practices on Growth, Phosphorus Uptake, and Yield of Maize in Controlled and Field-Based Studies in Relation to Arbuscular Mycorrhizal Fungi. Appl. Microbiol..

[24] Lahbouki S., Fernando A.L., Rodrigues C., Ben-Laouane R., Ait-El-Mokhtar M., Outzourhit A., Meddich A. (2023). Effects of Humic Substances and Mycorrhizal Fungi on Drought-Stressed Cactus: Focus on Growth, Physiology, and Biochemistry. Plants.

[25] Wanlin L.I., Yan X. (2024). Effects of Polystyrene Microplastics, Simulated Acid Rain and Arbuscular Mycorrhizal Fungi on the Growth of Trifolium Repens and Soil Microbial Community Composition. Pedosphere.

[26] Bisht A., Garg N. (2022). AMF Species Improve Yielding Potential of Cd Stressed Pigeonpea Plants by Modulating Sucrose-Starch Metabolism, Nutrients Acquisition and Soil Microbial Enzymatic Activities. Plant Growth Regul..

[27] Leventis G., Tsiknia M., Feka M., Ladikou E.V., Papadakis I.E., Chatzipavlidis I., Papadopoulou K., Ehaliotis C. (2021). Arbuscular Mycorrhizal Fungi Enhance Growth of Tomato under Normal and Drought Conditions, via Different Water Regulation Mechanisms. Rhizosphere.

[28] Besharati J., Shirmardi M., Meftahizadeh H., Ardakani M.D., Ghorbanpour M. (2022). Changes in Growth and Quality Performance of Roselle (*Hibiscus sabdariffa* L.) in Response to Soil Amendments with Hydrogel and Compost under Drought Stress. South African J. Bot..

[29] Lahbouki S., Ben-Laouane R., Outzourhit A., Meddich A. (2022). The Combination of Vermicompost and Arbuscular Mycorrhizal Fungi Improves the Physiological Properties and Chemical Composition of *Opuntia ficus-indica* under Semi-Arid Conditions in the Field. Arid L. Res. Manag..

[30] Rashtbari M., Hossein Ali A., Ghorchiani M. (2020). Effect of Vermicompost and Municipal Solid Waste Compost on Growth and Yield of Canola under Drought Stress Conditions. Commun. Soil Sci. Plant Anal..

[31] El Amerany F., Rhazi M., Wahbi S., Taourirte M., Meddich A. (2020). The Effect of Chitosan, Arbuscular Mycorrhizal Fungi, and Compost Applied Individually or in Combination on Growth, Nutrient Uptake, and Stem Anatomy of Tomato. Sci. Hortic..

[32] Paymaneh Z., Sarcheshmehpour M., Mohammadi H., Hesni M.A. (2023). Vermicompost and/or Compost and Arbuscular Mycorrhizal Fungi Are Conducive to Improving the Growth of Pistachio Seedlings to Drought Stress. Appl. Soil Ecol..

[33] Pant A., Radovich T.J.K., Hue N.V., Arancon N.Q. (2011). Effects of Vermicompost Tea (Aqueous Extract) on Pak Choi Yield, Quality, and on Soil Biological Properties. Compost Sci. Util..

[34] Erdal İ., Ekinci K. (2020). Effects of Composts and Vermicomposts Obtained from Forced Aerated and Mechanically Turned Composting Method on Growth, Mineral Nutrition and Nutrient Uptake of Wheat. J. Plant Nutr..

[35] Tammam A.A., Rabei Abdel Moez Shehata M., Pessarakli M., El-Aggan W.H. (2023). Vermicompost and Its Role in Alleviation of Salt Stress in Plants–I. Impact of Vermicompost on Growth and Nutrient Uptake of Salt-Stressed Plants. J. Plant Nutr..

[36] Bezabeh M.W., Haile M., Sogn T.A., Eich-Greatorex S. (2021). Yield, Nutrient Uptake, and Economic Return of Faba Bean (*Vicia faba* L.) in Calcareous Soil as Affected by Compost Types. J. Agric. Food Res..

[37] Lahbouki S., Ben-Laouane R., Anli M., Boutasknit A., Ait-Rahou Y., Ait-El-Mokhtar M., El Gabardi S., Douira A., Wahbi S., Outzourhit A. (2022). Arbuscular Mycorrhizal Fungi and/or Organic Amendment Enhance the Tolerance of Prickly Pear (*Opuntia ficus-indica*) under Drought Stress. J. Arid Environ..

[38] Ahmad A., Aslam Z., Ahmad M., Zulfiqar U., Yaqoob S., Hussain S., Niazi N.K., Gastelbondo M., Al-Ashkar I., Elshikh M.S. (2024). Vermicompost Application Upregulates Morpho-Physiological and Antioxidant Defense to Conferring Drought Tolerance in Wheat. Plant Stress.

[39] Lamaizi S., Meddich A., Boutasknit A., Anli M., Lahbouki S., El Fels L., Ouhdouch Y., Hafidi M. (2023). Application of Olive-Mill-Wastewater-Compost in Combination with Symbiotic Microorganisms Improves the Physiological, Biochemical Performance and Tolerance of Tomato (*Solanum lycopersicum)* Under Drought Stress. Gesunde Pflanz..

[40] Olayiwola V.A., Abiodun F.O. (2019). Effects of Arbuscular Mycorrhiza Fungi (*Glomus mossae*) and Compost on Early Growth Performance of Parkia Biglobosa. Curr. J. Appl. Sci. Technol..

[41] Benaffari W., Boutasknit A., Anli M., Ait-El-Mokhtar M., Ait-Rahou Y., Ben-Laouane R., Ben Ahmed H., Mitsui T., Baslam M., Meddich A. (2022). The Native Arbuscular Mycorrhizal Fungi and Vermicompost-Based Organic Amendments Enhance Soil Fertility, Growth Performance, and the Drought Stress Tolerance of Quinoa. Plants.

[42] Ouhaddou R., Ech-chatir L., Anli M., Ben-Laouane R., Boutasknit A., Meddich A. (2023). Secondary Metabolites, Osmolytes and Antioxidant Activity as the Main Attributes Enhanced by Biostimulants for Growth and Resilience of Lettuce to Drought Stress. Gesunde Pflanz..

[43] Dong S., Hu F., Alamusa, Ma Q., Liu Z. (2023). Arbuscular Mycorrhizal Fungi Enhance the Drought Resistance More Significantly of the Late-successional Psammophytes than That of the Early Ones. Restor. Ecol..

[44] Quiroga G., Erice G., Aroca R., Delgado-Huertas A., Ruiz-Lozano J.M. (2020). Elucidating the Possible Involvement of Maize Aquaporins and Arbuscular Mycorrhizal Symbiosis in the Plant Ammonium and Urea Transport under Drought Stress Conditions. Plants.

[45] Cavagnaro T.R. (2015). Biologically Regulated NutrientSupply Systems. Compost AndArbuscular Mycorrhizas-A Review. Adv. Agron..

[46] Liu M., Shen Y., Li Q., Xiao W., Song X. (2021). Arbuscular Mycorrhizal Fungal Colonization and Soil PH Induced by Nitrogen and Phosphorus Additions Affects Leaf C: N: P Stoichiometry in Chinese Fir (*Cunninghamia lanceolata*) Forests. Plant Soil.

[47] Li H., Zhang X., Hou X., Du T. (2021). Developmental and Water Deficit-Induced Changes in Hydraulic Properties and Xylem Anatomy of Tomato Fruit and Pedicels. J. Exp. Bot..

[48] Zimmermann J., Link R.M., Hauck M., Leuschner C., Schuldt B. (2021). 60-Year Record of Stem Xylem Anatomy and Related Hydraulic Modification under Increased Summer Drought in Ring-and Diffuse-Porous Temperate Broad-Leaved Tree Species. Trees.

[49] Li H., Hou X., Du T. (2023). Responses of Tomato Fruit Water Balance and Xylem Hydraulic Property of Pedicel and Calyx to Water Deficit and Salinity Stress. Environ. Exp. Bot..

[50] Kaur S., Suseela V. (2020). Unraveling Arbuscular Mycorrhiza-Induced Changes in Plant Primary and Secondary Metabolome. Metabolites.

[51] Hou L., Li M., Zhang C., Liu N., Liu X., Bo W., Pang X., Li Y. (2022). Comparative Transcriptomic Analyses of Different Jujube Cultivars Reveal the Co-Regulation of Multiple Pathways during Fruit Cracking. Genes.

[52] Zhang X., Yang H., Du T. (2024). Coupled Mechanisms of Water Deficit and Soil Salinity Affecting Tomato Fruit Growth. Agric. Water Manag..

[53] Jiaying M., Tingting C., Jie L., Weimeng F., Baohua F., Guangyan L., Hubo L., Juncai L., Zhihai W., Longxing T. (2022). Functions of Nitrogen, Phosphorus and Potassium in Energy Status and Their Influences on Rice Growth and Development. Rice Sci..

[54] Püschel D., Bitterlich M., Rydlová J., Bukovská P., Sudová R., Jansa J. (2023). Benefits in Plant N Uptake via the Mycorrhizal Pathway in Ample Soil Moisture Persist under Severe Drought. Soil Biol. Biochem..

[55] Khalediyan N., Weisany W., Schenk P.M. (2021). Arbuscular Mycorrhizae and Rhizobacteria Improve Growth, Nutritional Status and Essential Oil Production in *Ocimum Basilicum* and *Satureja Hortensis*. Ind. Crops Prod..

[56] Das D., Abbhishek K., Banik P., Bhattacharya P. (2021). A Valorisation Approach in Recycling of Organic Wastes Using Low-Grade Rock Minerals and Microbial Culture through Vermicomposting. Environ. Chall..

[57] Chen Q., Qu Z., Ma G., Wang W., Dai J., Zhang M., Wei Z., Liu Z. (2022). Humic Acid Modulates Growth, Photosynthesis, Hormone and Osmolytes System of Maize under Drought Conditions. Agric. Water Manag..

[58] Siles J.A., José M., González-Pérez J.A., Fernández-Pérez V., García-Díaz C., Moreno J.L., García C., Bastida F. (2024). Long-Term Restoration with Organic Amendments Is Clearer Evidenced by Soil Organic Matter Composition than by Changes in Microbial Taxonomy and Functionality. Appl. Soil Ecol..

[59] Okla M.K., Rubnawaz S., Dawoud T.M., Al-Amri S., El-Tayeb M.A., Abdel-Maksoud M.A., Akhtar N., Zrig A., Abdelgayed G., Abdelgawad H. (2022). Laser Light Treatment Improves the Mineral Composition, Essential Oil Production and Antimicrobial Activity of Mycorrhizal Treated *Pelargonium graveolens*. Molecules.

[60] Bi Y., Wu C., Wang S., Gao X., Xue C., Yang W., Li M., Xiao L., Christie P. (2022). Combined Arbuscular Mycorrhizal Inoculation and Loess Amendment Improve Rooting and Revegetation Post-Mining. Rhizosphere.

[61] Kaur H., Manna M., Thakur T., Gautam V., Salvi P. (2021). Imperative Role of Sugar Signaling and Transport during Drought Stress Responses in Plants. Physiol. Plant..

[62] Francesca S., Cirillo V., Raimondi G., Maggio A., Barone A., Rigano M.M. (2021). A Novel Protein Hydrolysate-Based Biostimulant Improves Tomato Performances under Drought Stress. Plants.

[63] Soussani F.E., Boutasknit A., Ben-Laouane R., Benkirane R., Baslam M., Meddich A. (2023). Arbuscular Mycorrhizal Fungi and Compost-Based Biostimulants Enhance Fitness, Physiological Responses, Yield, and Quality Traits of Drought-Stressed Tomato Plants. Plants.

[64] Khosravi Shakib A., Rezaei Nejad A., Khandan Mirkohi A., Kalate Jari S. (2019). Vermicompost and Manure Compost Reduce Water-Deficit Stress in Pot Marigold (*Calendula Officinalis* L. Cv. Candyman Orange). Compost Sci. Util..

[65] Ganugi P., Fiorini A., Tabaglio V., Capra F., Zengin G., Bonini P., Caffi T., Puglisi E., Trevisan M., Lucini L. (2023). The Functional Profile and Antioxidant Capacity of Tomato Fruits Are Modulated by the Interaction between Microbial Biostimulants, Soil Properties, and Soil Nitrogen Status. Antioxidants.

[66] Etesami H., Jeong B.R. (2021). Contribution of Arbuscular Mycorrhizal Fungi, Phosphate–Solubilizing Bacteria, and Silicon to P Uptake by Plant: A Review. Front. Plant Sci..

[67] Sandmann G. (2021). Diversity and Origin of Carotenoid Biosynthesis: Its History of Coevolution towards Plant Photosynthesis. New Phytol..

[68] Fanasca S., Colla G., Maiani G., Venneria E., Rouphael Y., Azzini E., Saccardo F. (2006). Changes in Antioxidant Content of Tomato Fruits in Response to Cultivar and Nutrient Solution Composition. J. Agric. Food Chem..

[69] El-Beltagi H.S., Mohamed H.I., Sofy M.R. (2020). Role of Ascorbic Acid, Glutathione and Proline Applied as Singly or in Sequence Combination in Improving Chickpea Plant through Physiological Change and Antioxidant Defense under Different Levels of Irrigation Intervals. Molecules.

[70] Talbi S., Rojas J.A., Sahrawy M., Rodríguez-Serrano M., Cárdenas K.E., Debouba M., Sandalio L.M. (2020). Effect of Drought on Growth, Photosynthesis and Total Antioxidant Capacity of the Saharan Plant *Oudeneya Africana*. Environ. Exp. Bot..

[71] Rodríguez-Arce E., Saldías M. (2021). Antioxidant Properties of Flavonoid Metal Complexes and Their Potential Inclusion in the Development of Novel Strategies for the Treatment against Neurodegenerative Diseases. Biomed. Pharmacother..

[72] Singh A., Roychoudhury A. (2023). Role of Phenolic Acids and Flavonoids in the Mitigation of Environmental Stress in Plants. Biology and Biotechnology of Environmental Stress Tolerance in Plants.

[73] Wang Y., Zhang W., Liu W., Ahammed G.J., Wen W., Guo S., Shu S., Sun J. (2021). Auxin Is Involved in Arbuscular Mycorrhizal Fungi-Promoted Tomato Growth and NADP-Malic Enzymes Expression in Continuous Cropping Substrates. BMC Plant Biol..

[74] Al Jaouni S., Selim S., Hassan S.H., Mohamad H.S.H., Wadaan M.A.M., Hozzein W.N., Asard H., AbdElgawad H. (2019). Vermicompost Supply Modifies Chemical Composition and Improves Nutritive and Medicinal Properties of Date Palm Fruits from Saudi Arabia. Front. Plant Sci..

[75] Lahbouki S., Anli M., El Gabardi S., Ait-El-Mokhtar M., Ben-Laouane R., Boutasknit A., Ait-Rahou Y., Outzourhit A., Wahbi S., Douira A. (2021). Evaluation of Arbuscular Mycorrhizal Fungi and Vermicompost Supplementation on Growth, Phenolic Content and Antioxidant Activity of Prickly Pear Cactus (*Opuntia ficus-indica*). Plant Biosyst. Int. J. Deal. All Asp. Plant Biol..

[76] Idris N.S., Khandaker M.M., Rashid Z.M., Majrashi A., Alenazi M.M., Nor Z.M., Mohd Adnan A.F., Mat N. (2023). Polyphenolic Compounds and Biological Activities of Leaves and Fruits of *Syzygium samarangense* Cv.‘Giant Green’at Three Different Maturities. Horticulturae.

[77] Lahbouki S., Ech-chatir L., Er-Raki S., Outzourhit A., Meddich A. (2022). Improving Drought Tolerance of *Opuntia ficus-indica* under Field Using Subsurface Water Retention Technology: Changes in Physiological and Biochemical Parameters. Can. J. Soil Sci..

[78] Phillips J.M., Hayman D.S. (1970). Improved Procedures for Clearing Roots and Staining Parasitic and Vesicular-Arbuscular Mycorrhizal Fungi for Rapid Assessment of Infection. Trans. Br. Mycol. Soc..

[79] Trouvelot A., Kough J.L., Gianinazzi-Pearson V., Gianinazzi S. (1986). Mesure Du Taux de Mycorhization VA d’un Système Radiculaire. Recherche de Méthode d’estimation Ayant Une Signification Fonctionnelle. Physiological and Genetical Aspects of Mycorrhizae.

[80] Tao H., Sun H., Wang Y., Wang X., Guo Y. (2023). Effects of Water Stress on Quality and Sugar Metabolism in ‘Gala’Apple Fruit. Hortic. Plant J..

[81] Dubois M., Gilles K.A., Hamilton J.K., Rebers P.A., Smith F. (1956). Colorimetric Method for Determination of Sugars and Related Substances. Anal. Chem..

[82] Bradford M.M. (1976). A Rapid and Sensitive Method for the Quantitation of Microgram Quantities of Protein Utilizing the Principle of Protein-Dye Binding. Anal. Biochem..

[83] Pastori P.L., FilgueiraS R.M.C., Oster A.H., Barbosa M.G., Silveira M.R.S.D.A., Paiva L.G.G. (2017). Postharvest Quality of Tomato Fruits Bagged with Nonwoven Fabric (TNT). Rev. Colomb. Cienc. Hortícolas.

[84] Zhou X., Huang W., Kong W., Ye H., Dong Y., Casa R. (2017). Assessment of Leaf Carotenoids Content with a New Carotenoid Index: Development and Validation on Experimental and Model Data. Int. J. Appl. Earth Obs. Geoinf..

[85] Roldán-Gutiérrez J.M., de Castro M.D.L. (2007). Lycopene: The Need for Better Methods for Characterization and Determination. TrAC Trends Anal. Chem..

[86] Adrian J., Peiró Esteban J.M. (2000). Análisis Nutricional de los Alimentos.

[87] Lee Y.P., Takahashi T. (1966). An Improved Colorimetric Determination of Amino Acids with the Use of Ninhydrin. Anal. Biochem..

[88] Singleton V.L., Rossi J.A. (1965). Colorimetry of Total Phenolics with Phosphomolybdic-Phosphotungstic Acid Reagents. Am. J. Enol. Vitic..

[89] Tohidi B., Rahimmalek M., Arzani A. (2017). Essential Oil Composition, Total Phenolic, Flavonoid Contents, and Antioxidant Activity of Thymus Species Collected from Different Regions of Iran. Food Chem..

[90] Aruwa C.E., Amoo S.O., Kudanga T. (2019). Extractable and Macromolecular Antioxidants of *Opuntia ficus-indica* Cladodes: Phytochemical Profiling, Antioxidant and Antibacterial Activities. South Afr. J. Bot..

[91] Watts S., Halliwell L. (1996). Essential Environmental Science: Methods & Techniques.

[92] Watts S., Halliwell L. (1996). Appendix 3–Detailed Field and Chemical Methods for Soil. Essential Environmental Science, Methods & Techniques.

[93] Wolf B. (1982). A Comprehensive System of Leaf Analyses and Its Use for Diagnosing Crop Nutrient Status. Commun. Soil Sci. Plant Anal..

[94] Aubert G. (1978). Methodes d’Analyses Des Sols: Documents de Travail Tous Droits Reserves.

[95] Olsen S.R., Sommers L.E. (1982). Phosphorus in Methods of Soil Analysis Part 2. Chem. Microbiol. Prop. Agron. Monogr..

