# Supplementary material for: Integration of Horse Manure Vermicompost Doses and Arbuscular Mycorrhizal Fungi to Improve Fruit Quality, and Soil Fertility in Tomato Field Facing Drought Stress
Source: Plants (Basel). 2024 May 23;13(11):1449. doi: 10.3390/plants13111449 (PMC11174961; doi:10.3390/plants13111449)
Supplement: Supplementary file 1 [file plants-13-01449-s001.zip › plants-2959730-supplementary.pdf]

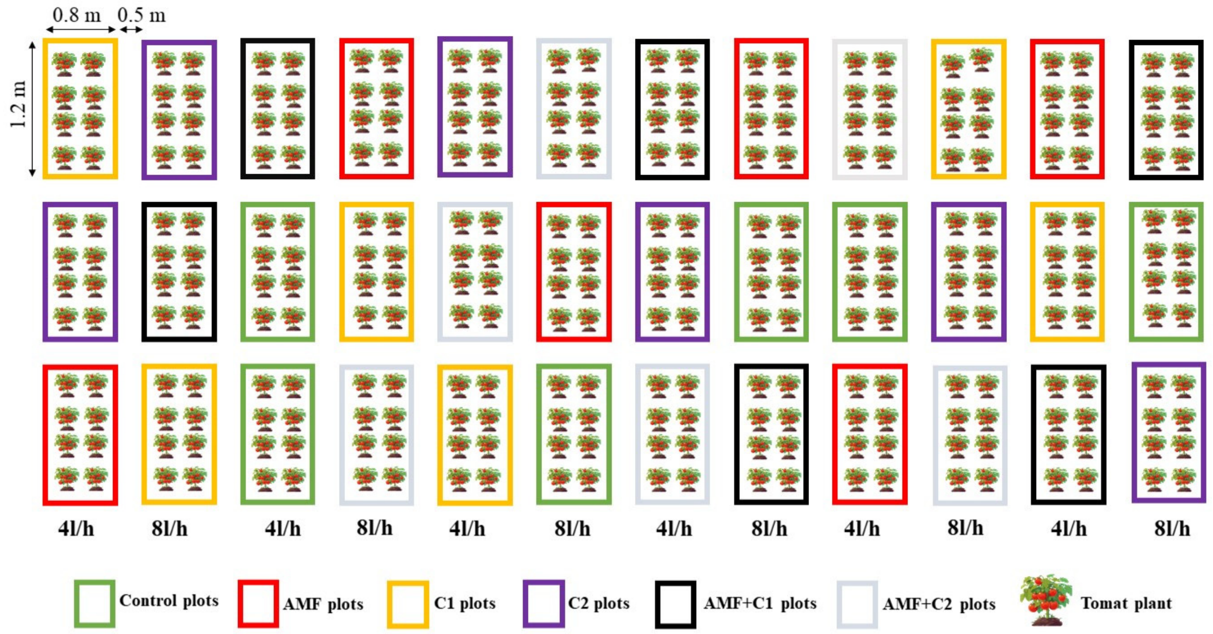

Figure S1: Experimental design map with, Control: control treatment; AMF plots: plants inoculated with AMF consortium; C1 plots: plants amended with 5 t ha^{-1} of organic amendment; C2 plots: plants amended with 10 t ha^{-1} of organic amendment; AMF + C1 plots: plants inoculated with the AMF consortium and amended in C1; AMF + C2 plots: plants inoculated with the AMF consortium and amended in C2.
